# Association between assisted reproductive technology and the risk of autism spectrum disorders in the offspring: a meta-analysis

**DOI:** 10.1038/srep46207

**Published:** 2017-04-07

**Authors:** Liang liu, Junwei Gao, Xie He, Yulong Cai, Lian Wang, Xiaotang Fan

**Affiliations:** 1 Department of Developmental Neuropsychology, School of Psychology, Third Military Medical University, Chongqing, 400038, China

## Abstract

The association between the use of assisted reproductive technology (ART) and autism spectrum disorder (ASD) risk in offspring has been explored in several studies, but the result is still inconclusive. We assessed the risk of ASD in offspring in relation to ART by conducting a meta-analysis. A literature search in PubMed, Embase, and Web of Knowledge databases through April 30, 2016 was conducted to identify all the relevant records. Risk ratios (RRs) and 95% confidence intervals (95%CIs) were computed to analyze the strength of association by using fixed- or random-effect models based on heterogeneity test in total and subgroup analyses. Analysis of the total 11 records (3 cohort studies and 8 case-control studies) revealed that the use of ART is associated with higher percentage of ASD (RR = 1.35, 95% CI: 1.09–1.68, *P* = 0.007). In addition, subgroup analyses based on study design, study location and study quality were conducted, and some subgroups also showed a statistically significant association. Our study indicated that the use of ART may associated with higher risk of ASD in the offspring. However, further prospective, large, and high-quality studies are still required.

Autism spectrum disorder (ASD) is a neurodevelopmental disorder characterized by impairments in social interaction and communication, together with restricted and repetitive behavior^1^. Despite much effort made on the therapy of ASD and care system, it is still a major public health problem around the world. The prevalence estimates of ASD have dramatically increased in the last decades, as high as 1 person in 132 worldwide in 2010^2^. Although the etiology of ASD remains uncertain, it is considered to be of multifactorial etiology, involving both genetic and environmental factors^3^,^4^. Therefore, there may be an important way to prevent ASD by identifying the causes of ASD. Recently, a number of studies have tried to explore the modifiable environmental risk factors of ASD. Among these risk factors, assisted reproductive technology (ART) was widely discussed because of its acceptability by more and more people.

Occurrence in pregnancy and birth factors have been indicated in the prevalence of ASD^5^,^6^,^7^. ART is used to achieve pregnancy and live birth through any procedure or medication trying to achieve pregnancy, including *in vitro* fertilization (IVF), zygote intrafallopian transfer (ZIFT), gamete intrafallopian transfer (GIFT), and artificial insemination among others. *In vitro* fertilization and intracytoplasmic sperm injection (ICSI) are standardized ART treatments, and over 5 million children have been born by these procedures worldwide^8^. As ART has become increasingly common, the developmental outcomes of these pregnancies have been of concomitant concern. It has been reported that the use of ART increased the total risk of congenital malformations by about one-third, with approximately a twofold increased risk of nervous systems defects^9^. Accumulated evidence has suggested that the use of ART may lead to an increased risk of birth defects, preterm delivery, low birth weight, and genetic imprinting disorders^10^,^11^, which may account for the development of ASD. Several observational and epidemiological studies have explored the relationship of the use of ART and ASD risk in offspring, but the results were inconsistent. A recent systematic review by Conti *et al*. concluded that there is no significant association ART and ASD in offspring based on 7 observational studies^12^. The investigators pointed out that the studies selected are heterogeneous in many aspects including study design, definitions of ART, data source, and analyzed confounders. However, a study from Sandin *et al*. found that fresh embryo transfer ART procedures using ICSI for male factor infertility were associated with increased risk of autistic disorder and intellectual disability in offspring compared with fresh embryo transfer procedures without ICSI^13^. This is consistent with the finding from Kissin *et al*., who claimed that the children from pregnancies using ICSI are also at higher risk for the incidence of autism compared with conventional IVF^14^. Notably, it may be particularly important to adjust for parental characteristics because users of ART are more likely than fertile individuals both to be of increased age and to have chromosomal abnormalities.

Although these studies have proven to be informative, no study to date has concurrently examined the general risk of ASD in offspring in relation to their exposure to ART versus natural conception. Therefore, we performed this study to systematically assess the association of the use of ART and ASD risk.

## Results

### Characteristics of eligible studies

**Figure 1:**
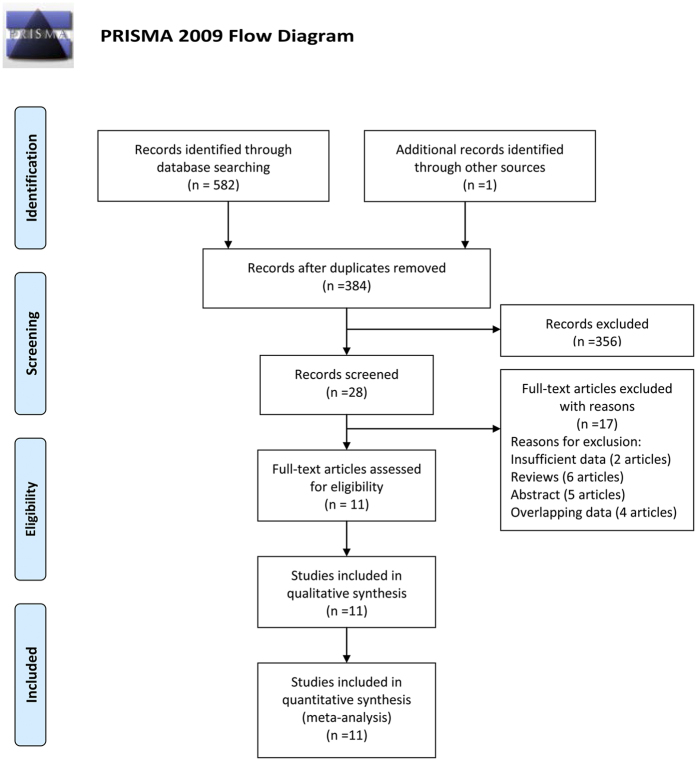
Flow diagram of study identification. From: Moher D, Liberati A, Tetzlaff J, Altman DG, The PRISMA Group (2009). Preferred Reporting Items for Systematic Reviews and Meta-Analyses: The PRISMA Statement.PLoS Med 6(7): e1000097. doi:10.1371/journal.pmed1000097. For more information, visit http://www.prisma-statement.org.

There were 572 records identified by using different search strategies in 3 databases (107 from PubMed, 265 from Embase, and 199 from Web of Knowledge) and 1 record by reference^15^. After removing 221 duplications and 323 unrelated records, 28 records were needed to further screen through full-text reading. Among the residual records, 13 records were excluded (2 records with insufficient data, 6 reviews, and 5 abstracts). Besides, there were 4 records^16^,^17^,^18^,^19^ excluded for overlapping data. Finally, 11 records^15^,^20^,^21^,^22^,^23^,^24^,^25^,^26^,^27^,^28^,^29^ (11 studies) were included in our meta-analysis (3 cohort studies and 8 case-control studies). The Flow diagram is shown in Fig. 1.

All of the included studies were published during 2006–2015, including 3 cohort studies and 8 case–control studies. Among them, 4 studies were performed in Europe, 4 in America and 3 in Asia. In all, a total of 8,161,225 patients were included, of them 46249 with ASD (0.56%). The effect estimates of studies were extracted or calculated by the original data and the effect estimates in 6 studies were adjusted. The results of the Newcastle–Ottawa Scale showed that 3 studies had the highest quality scores, 4 studies had moderate quality scores and 4 studies had low quality scores. The diagnosis in 10 studies was according to clinical evaluation and international coding. The characteristics of included studies are presented in Table 1 and Supplementary Table S1.

### Quantitative data synthesis

**Figure 2:**
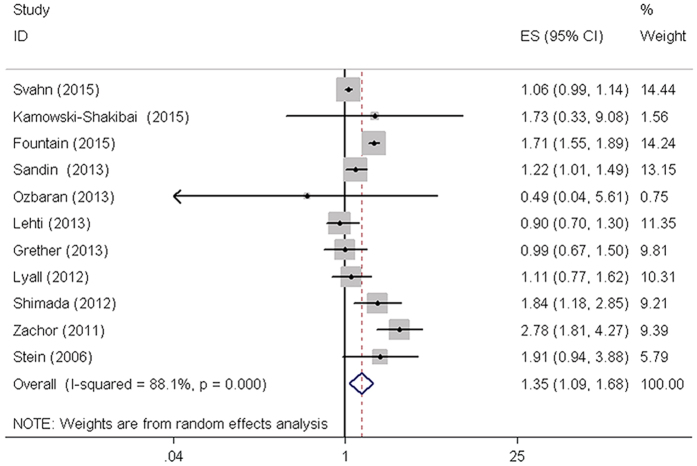
Forest plot of ASDs risk associated with ART using. ASDs, autism spectrum disorders; CI, confidence interval.

The effect of ART on the incidence of ASD was evaluated using the included studies. The overall results of our meta-analysis suggested that the use of ART may associated with higher percentage of ASD in children (RR = 1.35, 95% CI: 1.09–1.68, *P* = 0.007) (Fig. 2, Table 2). In addition, subgroup analyses were conducted based on study design, study location, study quality, singletons birth, multiple birth and preterm (<37 weeks). There was a significant association between ART and the risk of ASD in European and Asian populations. The pooled RR based on 3 high quality studies showed that pregnancy by ART was associated with an increased risk of ASD. The results were shown in Table 2. Importantly, we found preterm delivery (<37 weeks) appear to be mediating factors in the ART-autism association. The results of singletons birth, multiple birth and preterm (<37 weeks) subgroups analysis were presented in Supplementary Figure S1 and Supplementary Table S2.

### Heterogeneity analysis

Obvious between-study heterogeneity was shown in total analysis (*I*^*2*^* = *88.1%, *P* < 0.001) (Fig. 2, Table 2). To explore the source of between-study heterogeneity, we performed subgroup analyses based on study design, study location, and study quality. However, heterogeneity across studies did not decrease effectively. Subsequently, Galbraith plot was conducted to graphically evaluate the sources of heterogeneity. Three studies^20^,^22^,^28^ were outside the bounds in Galbraith plot (Supplementary Figure S2), which were identified as the primary sources of our between-study heterogeneity. Once the 3 studies were removed, the heterogeneity decreased effectively (*I*^*2*^* = *31.7%, *P* > 0.001). Although no obvious heterogeneity was detected again among the remaining studies, the corresponding pooled RR changed little (RR = 1.18, 95% CI: 1.03-1.34, *P* = 0.016) (Supplementary Figure S3). Therefore, ART may significantly increase the risk of ASD in children.

### Sensitivity analysis

Each study was removed sequentially to verify the effect on our results of an individual study. The result of sensitivity analysis suggested that no obvious changes were found after excluded any study. Therefore, no individual study in our meta-analysis affected our pooled RR value statistically and our results were reliable (Data not shown).

### Publication bias

**Figure 3:**
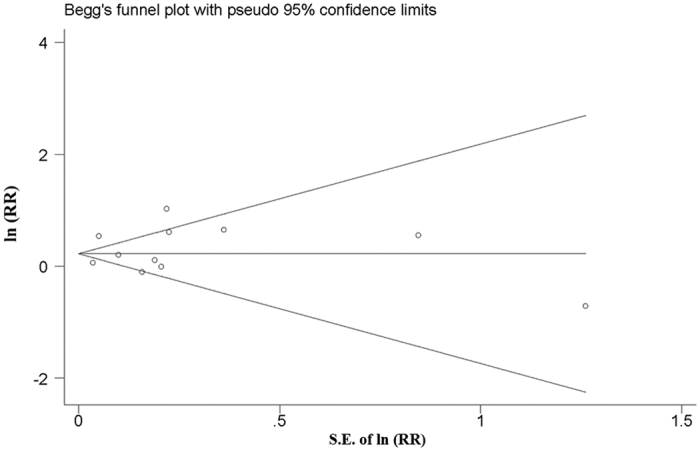
Funnel plots of studies examining the association between ASD and ART using. ASDs, autism spectrum disorders.

Both Egger’s and Begg’s methods were performed to explore the publication bias in our meta-analysis. Although slightly asymmetrical funnel plots were found in our results, there were no significant publication bias in our study (*P* = 0.755). The funnel plot was shown in Fig. 3.

## Discussion

To the best of our knowledge, this is the first meta-analysis to quantitatively evaluate the association between the use of ART and the risk of ASD in offspring. In this meta-analysis, 8,161,225 patients were included. With the accumulating evidence, we have enhanced statistical power to provide more precise and reliable risk estimates. The most-relevant heterogeneity moderators have been identified by subgroup analysis. These findings are important in showing that ART may be an independent risk factor for ASD.

Consistent with our results, the majority of prior work suggests increased risks of autism, or developmental delay, cerebral palsy, and imprinting disorders with use of ART^30^,^31^. However, a handful of studies reported that the use of ART did not decrease the child outcomes^32^,^33^. This divergence may be due to the too small sample size during previous studies. We also did subgroup analysis based on study design, study location, and study quality. We have found a significant association between ART and the risk of ASD in European and Asian populations. And the pooled RR based on 3 high quality studies also showed that pregnancy by ART was associated with an increased risk of ASD. However, for the limitation of data, we could not conduct a subgroup analysis according to the method of ART (IVF/ICSI) according to the rationale. Only 1 record^23^ has reported different findings for fresh embryo transfer ART procedures using, versus not using, ICSI. It showed a statistically significantly increased risk for ASD after ICSI using surgically extracted sperm with fresh embryos, compared with those born after IVF without ICSI with fresh embryos.

As we found that ART may be an independent risk factor for ASD. A possible mechanism linking ART and ASD is epigenetic changes induced by repeated hormone exposure, semen preparation, freezing of embryos and gametes, use of culture media, growth conditions for embryos, and delayed insemination. Epigenetic mechanisms such as defects in genetic imprinting are increasingly recognized to play an important role in several neuropsychiatric disorders, such as Rett and Fragile X syndromes, characterized by autistic-like features in some patients^34^. Melnyk *et al*. found abnormal methylation is particularly involved in ART imprinting disorders related to the context of ASD^35^. Experiments in animals have suggested that the various steps of the ART procedures such as superovulation, *in vitro* culture of oocytes or embryo, and IVF might be related to epigenetic defects in the embryos and offspring^36^. However, abnormal DNA methylation could not be consistently identified in IVF children^37^. It seemed that ART procedures together with etiological factors, including reduced fertility of the parents or the advanced maternal and paternal age, impact the epigenetic DNA methylation state. Recent studies has found epigenetic variability in the male or female germline, and occurrence of age-related DNA-methylation changes in a number of genes, and those changes may contribute to the increased ASD risk in offspring of older parents^38^,^39^,^40^.

For many environmental factors associated with ASD, some authors reported the maternal age, parental infertility, multiple birth and preterm delivery (<37 weeks). All included studies, with the exception of 4 studies, take into account possible confounders. Seven studies adjusted the estimates by many factors, including year of birth, infant’s gender, and mother’s education. Maternal age was a common flaw in studies of prenatal exposures, but among the included studies, only 1 study analyzed the influence of maternal age on ASD^22^. It found that the risk of autism of was higher for children born to mothers aged 20 to 34, but the effect was reduced to null for the mothers aged 35 years or older. The reasons behind this difference require further investigation. Besides, a recent study found a significant association between a general category of infertility medications and ASD among multiple births, but did not find an association among singleton births^41^. For comparison to this work, we tried to examine exposures stratified by singleton and multiple birth. We found that in 3 of the 11 studies, singletons analysis separately and showed that no significant associations were found between IVF and ASD among both in all offspring group and singleton subgroup^23^,^25^,^26^. And 2 of the 11 studies have conducted the subgroup analysis of multiple birth^22^,^26^, showing no significant difference between all offspring group and multiple birth subgroup. Importantly, there were two studies which have analyzed the influence of preterm delivery (<37 weeks) on the ART-autism association^22^,^23^. And the subgroup analysis result suggested preterm delivery may be a mediating factor in the ART-autism association. But the mechanisms behind this association require further investigation.

Besides, obvious heterogeneity was found among studies of ART and ASD risk. In our study, we noted that there were no significant associations between ART and ASD risk when obvious heterogeneity was found in subgroups and analysis by random (DerSimonian-Laird) effects model. Additionally, 3 studies contributing to the heterogeneity across all included studies potentially confounded the analyses. When we removed these 3 studies, the corresponding pooled RR value with very few changes was stable and reliable. Still, these estimates have to be viewed with caution due to heterogeneity. Moreover, potential publication bias had an important influence on the analysis, but little evidence of publication bias was observed. Since only eligible studies in English language were included in this meta-analysis, additional research in other populations is warranted to support generalization of the findings.

There are several limitations in our study. First, the meta-analysis was insufficiently performed due to the limited number of studies, which restrict the strength and quality of evidence. Thereby, more studies should be included in future reviews, to provide further support for our results. Second, residual confounding is a concern. The complexity of ART treatment renders the identification of individual risk factors extremely challenging. Uncontrolled or unmeasured risk factors have the potential to produce biases. It is very difficult to obtain information about an individual aspect of ART treatment and its association with risk of ASD, the possibility cannot be ruled out that residual confounding affected the results. Third, in the present study, we cannot explain the specific biological mechanisms underlying the relationship between using ART and the risk of ASDs. Meanwhile, it is still difficult to rule out the effects of factors related to the underlying subfertility.

In summary, ART was associated with a significantly greater risk of ASD in the offspring. ART is likely to be an independent risk factor of ASD in offspring. Because the number of available studies is still limited, our findings should be taken with caution. More studies, in particular population-based prospective cohort studies, are needed to verify the impact of ART on ASD risk in children. In addition, to understand the strength of association, future studies exploring the underlying molecular mechanisms are needed.

## Materials and Methods

This meta-analysis and systematic review protocol has been follow the PRISMA (Preferred Reporting Items for Systematic Reviews and Meta-Analyses) statement.

### Publication Search

All published articles were searched in the PubMed, Embase, and Web of Knowledge databases up to April 30, 2016. Key words were identified as followed: “autism”, “autistic”, “asperger syndrome” or “pervasive development disorders”; and “oocyte”, “fertilization”, “infertility”, “assisted reproductive technologies”, “intracytoplasmic sperm injection”, or “*in vitro* fertilization”. The search process was conducted by two independent investigators. All articles were retrieved and their references were checked to avoid missing other relevant articles. If data were not included in the original articles, we would contact related authors to obtain them.

### Inclusion and Exclusion Criteria

The inclusion criteria were as follows: (1) studies evaluating the association between ART and autism; (2) case–control or cohort studies; and (3) studies from which the effect estimates could be extracted or calculated from available data. The exclusion criteria were as follows: (1) studies with insufficient data to calculate or extract effect estimates; (2) case report, review, comment, abstract, or animal studies. For studies with overlapping samples, only the one with the largest sample size was included.

### Data Extract

Two independent researchers conducted data extraction to ensure the reliability of the results. Disagreement was resolved by discussion. The information of studies meeting the inclusion criteria was extracted using a standardized extraction form. Relevant information included the first author’s name, publication year, study country, study design, study period, diagnosis, data source, type of ART, the number of cases and controls, effect estimates and their corresponding 95% CI, and adjusted factors in the data analysis. If the effect estimates were not adjusted, we extracted a crude effect estimate. When the effect estimates were not presented in original studies, we calculate odds ratios or relative risk according to the data presented in the study.

### Assessment of study quality

The quality of studies was evaluated by 2 independent reviewers according to the Newcastle–Ottawa Scale, which contains 3 dimensions: selection, comparability, and exposure or outcome for case–control and cohort studies, respectively. Eight items were included to assess the quality of studies with a 9-star system. When the quality score of one study was greater than or equal to 7, we defined it as a high quality study, and we defined 0–3 stars and 4–6 stars, as low and moderate quality study, respectively^42^.

### Statistical analysis

Since the absolute risk of autism was low, relative risks and their corresponding 95% confidence interval were used as summary statistics to evaluate the association between maternal ART and the risk of autism in our meta-analysis, and the other measures of association were expected to yield similar estimates of relative risk. Z-test was conducted to assess the statistical significance of pooled RRs. Besides, the results were combined using a generic inverse variance random-effects model (DerSimonian and Laird) to calculate weights, where the weights depend on both within-study variance and the estimated between-study variance for random effects model. In addition to total analysis, we also carried out subgroup analyses based on study design, study location, and study quality. I-squared (I^2^) statistic and Chi-square based Q-test were used to investigate the heterogeneity across studies^43^. The selection of effects models was according to our heterogeneity test: *P > *0.10 for the Q-test and I^2^ values less than 50% suggested no obvious heterogeneity across studies and a fixed (Mantel-Haenszel) effects model was applied; otherwise, a random (DerSimonian-Laird) effects model was used^44^. In addition, Galbraith plots were used to further explore the source of between-study heterogeneity. We also performed sensitive analysis by removing each included study in sequence to assess the stability of our results. Publication bias was evaluated via a funnel plot using both Egger’s^45^ and Begg’s^46^ methods. Statistical significance was defined when a p-value < 0.05. All statistical analyses were performed using STATA 12.0 software (StataCorp, College Station, TX, USA).

## Additional Information

**How to cite this article:** liu, L. *et al*. Association between assisted reproductive technology and the risk of autism spectrum disorders in the offspring: a meta-analysis. *Sci. Rep.* **7**, 46207; doi: 10.1038/srep46207 (2017).

**Publisher's note:** Springer Nature remains neutral with regard to jurisdictional claims in published maps and institutional affiliations.

## Supplementary Material

Supplementary Information

## Figures and Tables

**Table 1:** Characteristics of the studies included in the meta-analysis.

Author (year)	Country	Diagnosis	Study design	Source of the study population	Study period	ART type	Outcome	case/control	RR(95%CI)	Adjusted factors	methodological quality
Svahn *et al*. (2015)^20^	Denmark	ICD-8/ICD-10	cohort	The computerized Civil Registration System	1969–	NA	ASD	2058/2410663	1.06(0.99, 1.14)	year of birth, birth order, sex, maternal age at birth, paternal age at birth and parental history of mental disorder	8
Kamowski-Shakibai *et al*. (2015)^21^	USA	NA	case-control	Children in the New York tri-state area	NA	ART	ASD	8/155	1.73(0.33, 9.08)	NA	2
Fountain *et al*. (2015)^22^	USA	DSM-IV/code 299.0	cohort	The California Birth Master Files for 1997–2007, the California DDS autism caseload records for 1997–2011, and the Centers for Disease Control and Prevention’s National ART Surveillance System for live births for 1997–2007	1997–2007	ART	ASD	31243/5529810	1.71(1.55, 1.89)	year of birth, infant’s gender, and mother’s education and race	6
Sandin *et al*. (2013)^23^	Sweden	ICD-9/ICD-10	cohort	Swedish national registers	1982–2009	IVF with ICSI or without ICSI	autistic disorder	6959/2541125	1.22(1.01, 1.49)	sex, attained age and birth year	9
Özbaran *et al*. (2013)^24^	Turkey	DSM-IV/ADSI/WISC-R	case-control	The outpatient clinic of Child and Adolescent Psychiatry Department of EUSM	NA	ART	Autism	3/67	0.49(0.04, 5.61)	NA	3
Lehti *et al*. (2013)^25^	Finland	ICD-9/ICD-10	case-control	The Finnish Hospital Discharge Register	1991–2007	IVF	ASD	4164/16582	0.9(0.70, 1.30)	maternal age and SES, gestational age and parity	8
Grether *et al*. (2013)^26^	USA	ICD-9	case-control	A KPNC facility	1995–2002	NA	ASD	349/1847	0.99(0.67, 1.50)	maternal and paternal age, maternal race, maternal education, baby sex, gestational age, birth year, and birth facility	6
Lyall *et al*. (2012)^27^	USA	ADI-R	case-control	Participants in the Nurses’ Health Study II	1989–	ART	ASD	507/2529	1.11(0.77, 1.62)	maternal and paternal age, race, income, and birth order	6
Shimada *et al*. (2012)^15^	Japan	DSM-IV-TR	case-control	University of Tokyo Hospital/General Population of Tokyo	2006–2009	IVF; ICSI	ASD	467/100118	1.84(1.18, 2.85)	NA	2
Zachor *et al*. (2011)^28^	Israel	DSM-IV-TR	case-control	Large Israel population from infant registry of Rabin Medical Center	1995–2002	IVF and ICSI	ASD	285/53080	2.78(1.81, 4.27)	NA	2
Stein *et al*. (2006)^29^	Israel	ICD 8/DSM III/IV	case-control	ALUT center of Tel Aviv	1970–1998	Infertility requiring medical intervention	autism	206/152	1.91(0.94, 3.88)	NA	5

NA: no avalible; DDS: Department of Developmental Services; EUSM: Ege University School of Medicine.

**Table 2:** Summary of meta-analysis results.

Groups	Studies	Test of association				Heterogeneity		
		RR[95%CI]	p value	Model	Z	Χ^2^	p value	*I*^2^(%)
Total studies	11	1.35[1.09–1.68]	0.007	RE^1^	2.7	83.76	0	88.1
Subgroup analyses								
study design								
cohort	3	1.30[0.93–1.83]	0.127	RE	1.52	59.36	0	96.6
case-control	8	1.40[0.99–1.98]	0.06	RE	1.89	24.15	0.001	71
scores								
high	3	1.07[1.00–1.14]	0.044	FE^2^	2.02	3.02	0.221	33.7
moderate	4	1.36[0.98–1.90]	0.07	RE	1.84	11.06	0.01	72.9
low	4	2.20[1.63–2.97]	0	FE	5.16	3.27	0.352	8.3
region								
America	4	1.30[0.91–1.87]	0.149	RE	1.44	10.85	0.013	72.4
Europe	3	1.07[1.00–1.14]	0.044	FE	2.02	3.02	0.221	33.7
Asia	4	2.17[1.64–2.87]	0	FE	5.42	3.33	0.343	10
after removing three studies	8	1.18[1.03–1.34]	0.016	FE	2.41	10.25	0.175	31.7

RE: random effects; FE: fixed effects.
