# Supplementary material for: Association between assisted reproductive technology and the risk of autism spectrum disorders in the offspring: a meta-analysis
Source: Sci Rep. 2017 Apr 7;7:46207. doi: 10.1038/srep46207 (PMC5384197; doi:10.1038/srep46207)
Supplement: Supplementary Information [file srep46207-s1.pdf]

Supplementary information

Association between assisted reproductive technology and the risk of autism spectrum disorders in the offspring: a meta-analysis

Liang liu, Junwei Gao, Xie He, Yulong Cai, Lian Wang, Xiaotang Fan

Department of Developmental Neuropsychology, School of Psychology, Third Military Medical University, Chongqing, 400038, China

Liang Liu and Junwei Gao contributed equally to this work.

*Address correspondence to：

Xiaotang Fan: Department of Developmental Neuropsychology, Third Military Medical University, Chongqing 400038, PR China.

Supplemental Figures and Figure Legends:

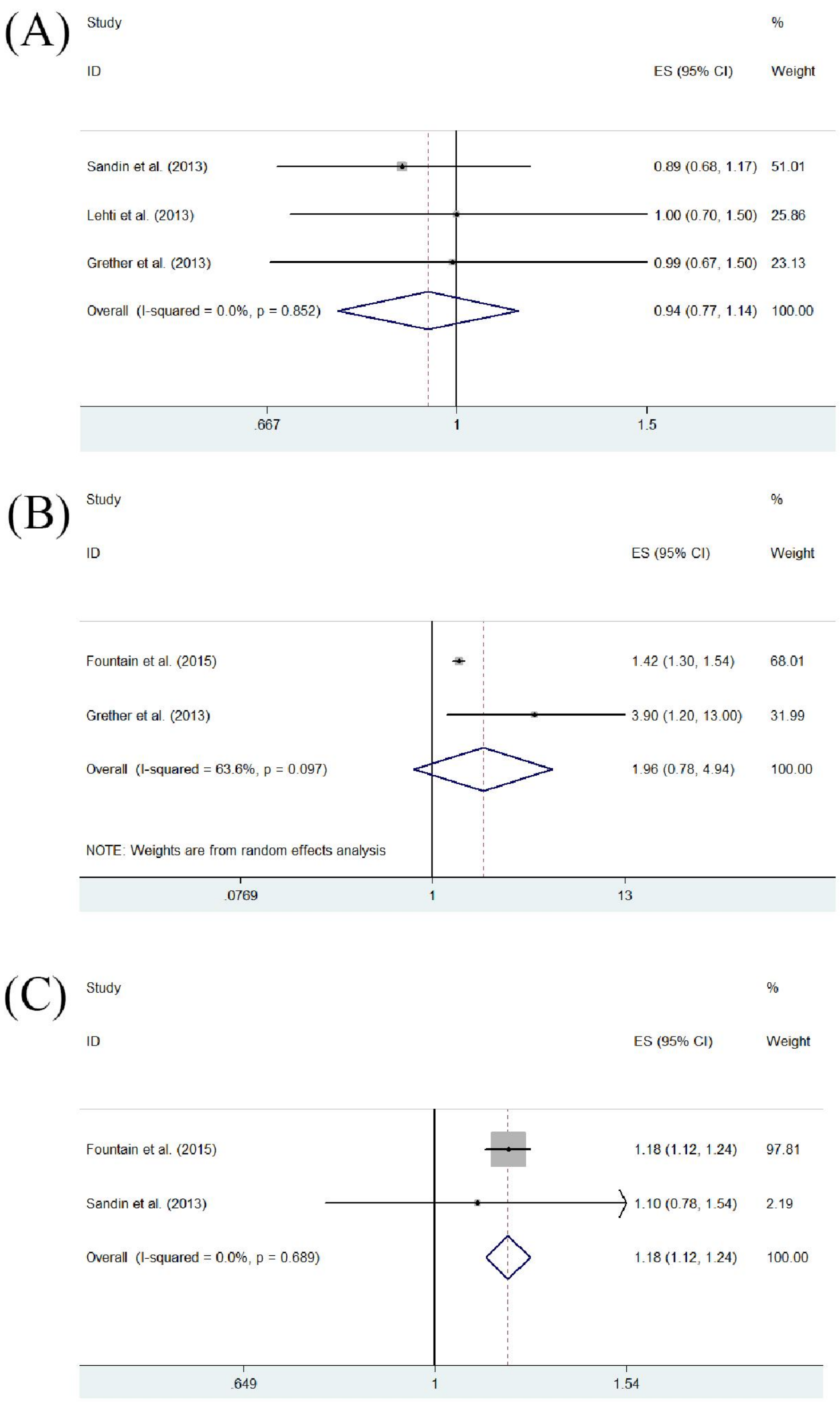

Supplementary Figure. S1. Forest plot of subgroups analysis by singletons birth **(A)**, multiple birth **(B)** and preterm (<37 weeks) **(C)**. CI, confidence interval

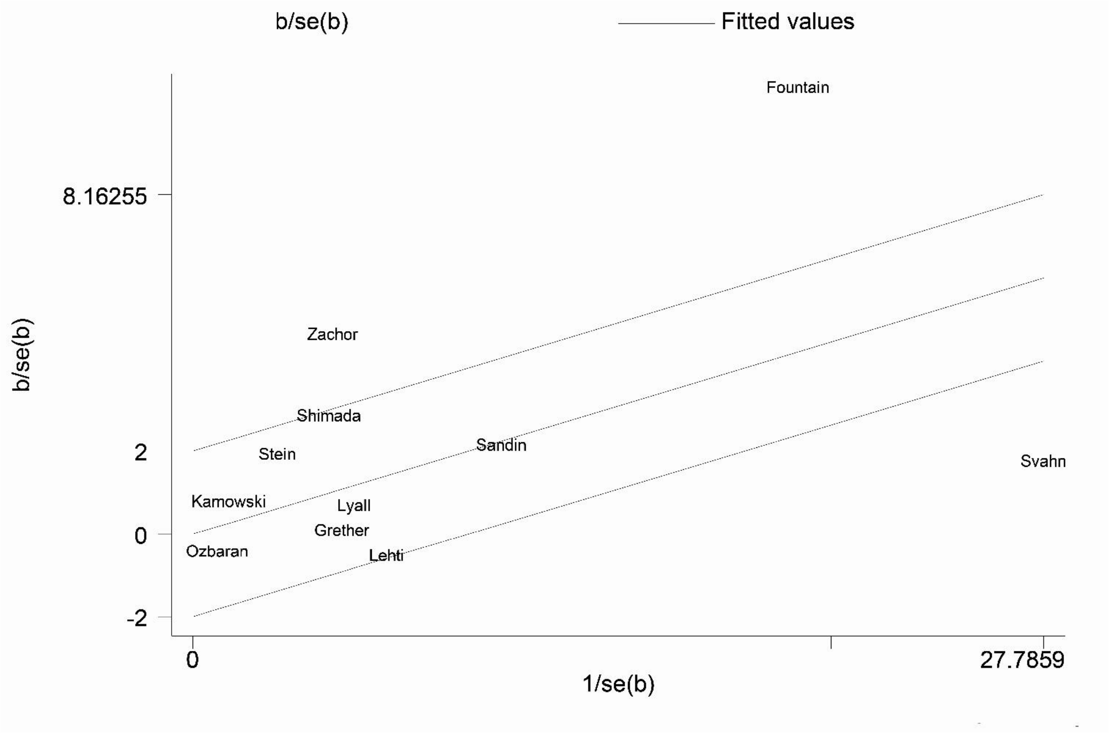

Supplementary Figure S2. Galbraith plot of ART using and the risk of ASDs among the children. ASDs, autism spectrum disorders.

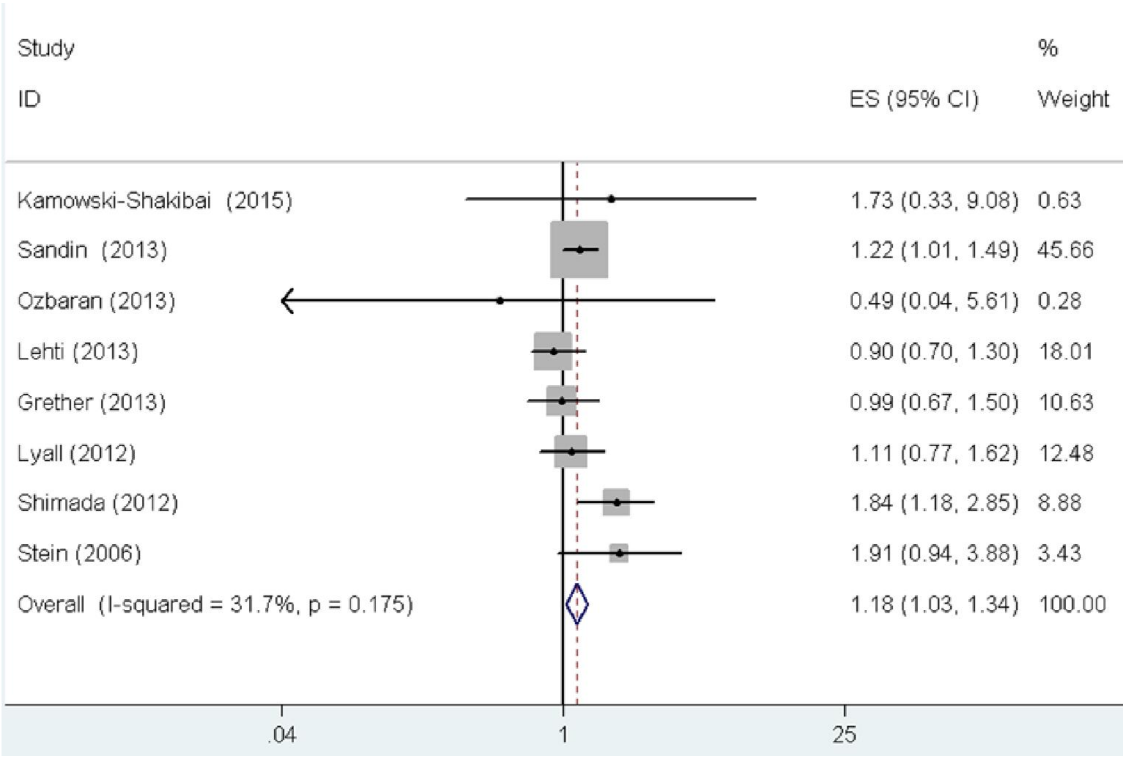

Supplementary Figure S3. Forest plot of ASDs risk associated with ART using after removing three studies. ASDs, autism spectrum disorders; CI, confidence interval.

Supplementary Table S1. The details about subgroups analysis for each study

Author (year)	Mothers' age <35 year old	Mothers' age >35 year old	Singletons birth	multiple birth	Preterm (<37 weeks)
Svahn et al. (2015)[1]	NA	NA	NA	NA	NA
Kamowski-Shakibai et al. (2015)[2]	NA	NA	NA	NA	NA
Fountain et al. (2015)[3]	1.21 (1.01, 1.45)	1.0 (0.88, 1.14)	NA	1.42 (1.30, 1.54)	1.18 (1.12, 1.24)
Sandin et al. (2013)[4]	NA	NA	0.89 (0.68, 1.17)	NA	1.10 (0.78, 1.54)
Özbaran et al. (2013)[5]	NA	NA	NA	NA	NA
Lehti et al. (2013)[6]	NA	NA	1.0 (0.7, 1.5)	NA	NA
Grether et al. (2013)[7]	NA	NA	0.99 (0.67, 1.5)	3.9 (1.2, 13)	NA
Lyall et al. (2012)[8]	NA	NA	NA	NA	NA
Shimada et al. (2012)[9]	NA	NA	NA	NA	NA
Zachor et al. (2011)[10]	NA	NA	NA	NA	NA
Stein et al. (2006)[11]	NA	NA	NA	NA	NA

NA: no avalible;

Supplementary Table S2. Summary of subgroups meta-analysis results.

		Test of association			Heterogeneity			
**Subgroup Groups**	**Studies**	**RR[95%CI]**	**p value**	**Model**	**Z**	**X^2**	**p value**	**I^2(%)**
Singletons birth	3	0.94[0.77-1.14]	0.153	FE	0.62	0.32	0.852	0
multiple birth	2	1.96[0.78-4.94]	0.532	RE	1.43	2.75	0.097	0.636
Preterm(<37 weeks)	2	1.18[1.12-1.24]	0	FE	6.39	0.16	0.689	0

RE: random effects; FE: fixed effects;

Reference:

1 Svahn, M. F. *et al.* Mental disorders in childhood and young adulthood among children born to women with fertility problems. *Human Reproduction* **30**, 2129-2137, (2015).

2 Kamowski-Shakibai, M. T., Magaldi, N. & Kollia, B. Parent-reported use of assisted reproduction technology, infertility, and incidence of autism spectrum disorders. *Research in Autism Spectrum Disorders* **9**, 77-95, (2015).

3 Fountain, C. *et al.* Association Between Assisted Reproductive Technology Conception and Autism in California, 1997-2007. *American Journal of Public Health* **105**, 963-971, (2015).

4 Sandin, S., Nygren, K. G., Iliadou, A., Hultman, C. M. & Reichenberg, A. Autism and mental retardation among offspring born after in vitro fertilization. *JAMA - Journal of the American Medical Association* **310**, 75-84, (2013).

5 Özbaran, B. *et al.* Psychiatric evaluation of children born with assisted reproductive technologies and their mothers: A clinical study. *Noropsikiyatri Arsivi* **50**, 59-64, (2013).

6 Lehti, V. *et al.* Autism spectrum disorders in IVF children: A national case-control study in Finland. *Human Reproduction* **28**, 812-818, (2013).

7 Grether, J. K. *et al.* Is infertility associated with childhood autism? *Journal of Autism and Developmental Disorders* **43**, 663-672, (2013).

8 Lyall, K., Pauls, D. L., Spiegelman, D., Santangelo, S. L. & Ascherio, A. Fertility therapies, infertilityand autism spectrum disorders in the Nurses' Health Study II. *Paediatric and Perinatal Epidemiology* **26**, 361-372, (2012).

9 Shimada T *et al.* Parental age and assisted reproductive technology in autism spectrum disorders, attention deficit hyperactivity disorder, and Tourette syndrome in a Japanese population. *Res Autism Spec Dis*, 500-507, (2012).

10 Zachor, D. A. & Ben Itzchak, E. Assisted reproductive technology and risk for autism spectrum disorder. *Research in Developmental Disabilities* **32**, 2950-2956, (2011).

11 Stein, D., Weizman, A., Ring, A. & Barak, Y. Obstetric complications in individuals diagnosed with autism and in healthy controls. *Comprehensive psychiatry* **47**, 69-75, (2006).
